# A Case of Surgery for Myxoma in the Inferior Vena Cava Using Deep Hypothermic Circulatory Arrest

**DOI:** 10.3400/avd.cr.24-00118

**Published:** 2025-03-25

**Authors:** Masato Hayama, Kayo Wakamatsu, Yuko Teratani, Yuki Kunitomo, Chihaya Ito, Masayuki Shimizu, Hiromitsu Teratani, Yuta Sukehiro, Masato Furui, Mizuki Sumi, Mau Amako, Yoshio Hayashida, Go Kuwahara, Hideichi Wada

**Affiliations:** 1 Department of Cardiovascular Surgery, Fukuoka University Hospital, Fukuoka, Fukuoka, Japan; 2 Department of Cardiovascular Surgery, Hakujuji Hospital, Fukuoka, Fukuoka, Japan; 3 Hakujuji Rehabilitation Hospital, Fukuoka, Fukuoka, Japan

**Keywords:** inferior vena cava tumor, myxoma, deep hypothermic circulatory arrest

## Abstract

We experienced a case in which a myxoma in the inferior vena cava (IVC) was surgically removed along with the IVC using deep hypothermic circulatory arrest. A 42-year-old female with no subjective symptoms was incidentally found to have a mass in the IVC at the junction of the hepatic veins on contrast-enhanced computed tomography. Ultrasonography revealed a mobile tumor attached to the junction of the hepatic veins. Surgery was performed via median sternotomy and laparotomy. Cardiopulmonary bypass and deep hypothermic circulatory arrest were utilized to safely operate.

## Introduction

When resecting a tumor in the inferior vena cava (IVC), it is very important to ensure a good surgical field for complete tumor resection. In addition, the need for intraoperative pulmonary embolization prevention and venous reconstruction must be considered. The method of extracorporeal circulation as an adjunct to tumor resection is controversial, but we report the successful resection of an IVC tumor bordering a hepatic vein bifurcation using hypothermic circulatory arrest.

## Case Report

Patient: 42-year-old female

Chief complaint: Abdominal pain

Present illness: The patient has experienced recurrent diverticulitis since 2012. In August 2024, a contrast-enhanced computed tomography (CT) scan was performed because of abdominal pain. The CT tomography revealed an 18 × 14 mm mass in the IVC and ultrasound examination confirmed a movable mass, leading to a referral for surgical treatment.

Medical history: History of uterine fibroid surgery and ovarian cyst surgery.

Preoperative physical examination: The abdomen was soft, and no ascites or tenderness was noted. No edema was observed in either lower extremity. There were also no features suspicious of Carney syndrome.

Family history: No special findings.

Preoperative blood test results: No significant abnormalities, including tumor markers, were found.

**Figure 1:**
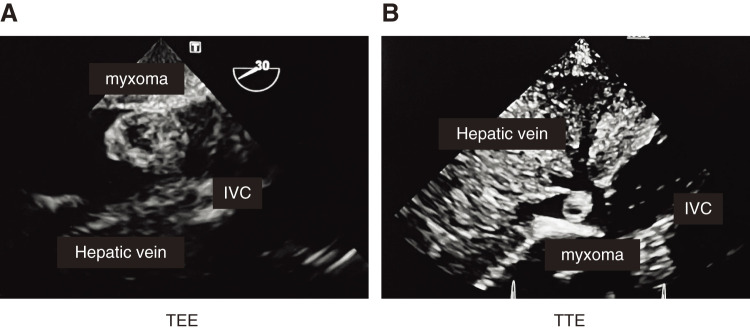
Fig. 1 The mass showed low echogenicity and hypovascular characteristics. (**A**) Transthoracic echocardiography. (**B**) Transesophageal echocardiography.

Ultrasound findings: A pedunculated movable mass measuring 18 × 14 mm was observed in the IVC at the entrance to the hepatic vein (**Fig. 1**).

Transesophageal echocardiography findings: The mass showed low echogenicity and hypovascular characteristics (**Fig. 1**).

**Figure 2:**
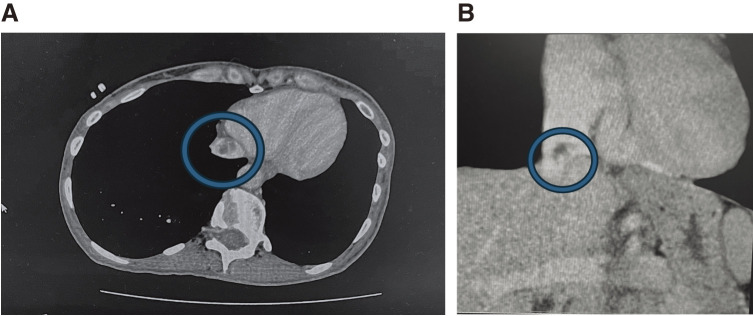
Fig. 2 A tumor was identified at the level of the IVC at the entrance of the hepatic vein, with evidence of blood flow within the tumor. (**A**) Axial section. (**B**) Coronal section. IVC: inferior vena cava

Abdominal contrast-enhanced CT findings: A tumor was identified at the level of the IVC at the entrance of the hepatic vein, with evidence of blood flow within the tumor (**Fig. 2**).

**Figure 3:**
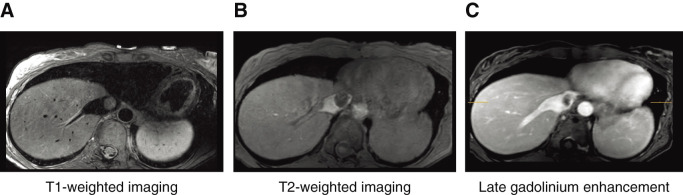
Fig. 3 (**A**) T1-weighted imaging showed a low signal with no fat components. (**B**) T2-weighted imaging showed a slightly heterogeneous, mildly high signal. (**C**) A faint enhancement effect was observed, predominantly at the margins.

Abdominal contrast-enhanced MRI findings: T2-weighted imaging showed a slightly heterogeneous, mildly high signal, whereas T1-weighted imaging showed a low signal with no fat components. By contrast, a faint enhancement effect was observed, predominantly at the margins (**Fig. 3**).

**Figure 4:**
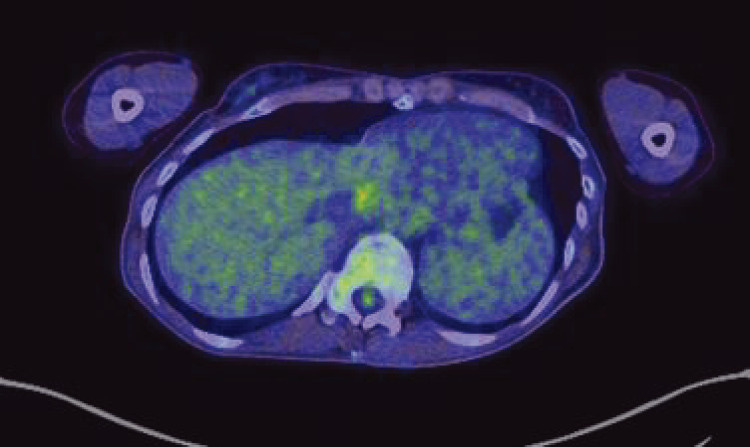
Fig. 4 The tumor showed FDG uptake (e/d SUVmax = 3.02/3.39), indicating significant accumulation. FDG: fluorodeoxyglucose

PET-CT findings: The tumor showed fluorodeoxyglucose (FDG) uptake (e/d SUVmax = 3.02/3.39), indicating significant accumulation. No other findings suggestive of metastasis or primary tumors were noted (**Fig. 4**).

Based on this, it was diagnosed as an IVC tumor, and a decision to perform the surgery was made. Owing to the nonspecific nature of the tumor location, we could not determine whether it was benign, malignant, or a thrombus during preoperative examinations. If it is a malignant tumor such as leiomyosarcoma or angiosarcoma, extensive resection of the IVC is necessary. Additionally, the tumor was attached at the entrance of the hepatic vein, making occlusion of the hepatic vein difficult and requiring liver handling. Thus, we decided to use a cardiopulmonary bypass for manipulation within the IVC and employ deep hypothermic circulatory arrest during the surgery.

### Surgical findings

**Figure 5:**
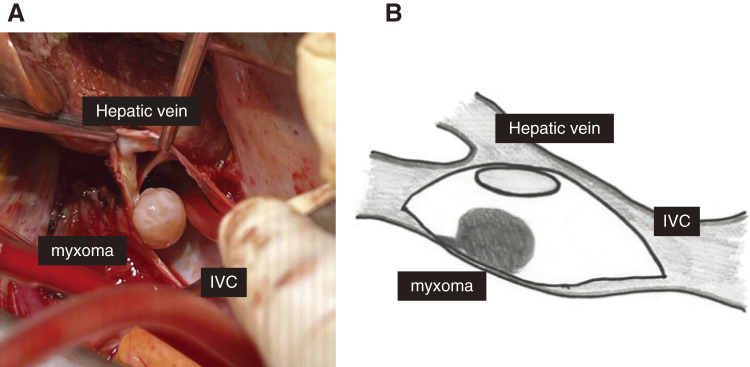
Fig. 5 (**A**) The tumor measured 14 × 12 mm, and the stalk was attached to the IVC at the entrance of the hepatic vein. (**B**) The scheme of the tumor and the vessels. IVC: inferior vena cava

Median sternotomy and laparotomy were performed. We incised the pericardium and diaphragm and exposed the IVC in the abdominal cavity. The mesentery attached to the liver was incised, the left lobe of the liver was retracted to the right, and the IVC and hepatic veins were dissected as much as possible. A cannula was inserted into the ascending aorta for arterial blood flow, a venous cannula was placed in the superior vena cava and right atrium, and a vent tube was inserted from the right upper pulmonary vein to the left ventricle along with a myocardial protection cannula in the ascending aorta. Cooling was initiated. After adequate cooling (pharyngeal temperature 18°C, bladder temperature 22°C, and rectal temperature 22°C), the circulatory arrest was initiated, and the IVC was incised. Upon observation of the IVC, the tumor was pedunculated and had a glossy surface. The tumor measured 14 × 12 mm, and the stalk was attached to the IVC at the entrance of the hepatic vein (**Fig. 5A**). We excised the entire section of the IVC to which the stalk was attached and submitted it for rapid intraoperative pathological diagnosis. Because the defect in the IVC was not large, closure was performed using a continuous suture with 4-0 Prolene (Johnson & Johnson, New Brunswick, NJ, USA) (**Fig. 5B**). Before closure, circulation was restarted, and the air was evacuated from the IVC. While warming the patient, we confirmed the rapid intraoperative pathological results, which showed no malignant findings. Once rewarming was complete, we weaned off the cardiopulmonary bypass, performed standard closure of the abdomen and chest, and completed the surgery.

### Surgery duration

294 min, cardiopulmonary bypass time: 99 min, aortic occlusion time: 24 min, cooling time: 51 min, circulatory arrest time: 14 min.

### Histopathological findings

The specimen was a circumscribed nodular lesion composed of bland spindle-shaped cells with abundant capillaries and myxoid stroma. No nuclear atypia, mitotic figures, or necrotic foci were observed. The possibility of a myxoma cannot be ruled out.

### Postoperative course

The patient was extubated the following day, recovered well, and was discharged on post operative day (POD) 14.

## Discussion

Myxomas arising from the right atrium are uncommon, constituting approximately 15%–20% of cases. Typically, these tumors arise from the atrial septum and rarely originate in the IVC. There are few reported cases, but many have arisen from the eustachian valve or its junction with the right atrium.^1–8)^ In this case, the tumor did not originate from the IVC at the entrance of the peripheral hepatic vein, which is rare. Symptoms have been reported to range from asymptomatic to exercise-induced dyspnea and Budd-Chiari syndrome.^1–8)^

When a tumor is located in the IVC, it is essential to secure a bloodless field and excise it safely. If occlusion is feasible, this is the simplest and the best approach. However, in this case, the tumor was attached to the entrance of the hepatic vein, making occlusion difficult. Although we considered using the Pringle maneuver to occlude the hepatic blood flow, we judged that if the tumor was malignant, necessitating extensive resection and reconstruction of the IVC, the risk of liver ischemia would increase with prolonged occlusion. Therefore, we opted for a cardiopulmonary bypass and induced deep hypothermic circulatory arrest after sufficient cooling. Deep hypothermic circulatory arrest allowed us to obtain a completely bloodless field solely through circuit suction, which also provided sufficient time. Fortunately, the tumor was a myxoma, allowing careful observation of the attachment site, which was meticulously excised without leaving any remnants. The resection range of the IVC was small enough to permit direct anastomotic closure.

## Conclusion

Myxomas in the IVC outside the junction with the eustachian valve or right atrium are extremely rare. In challenging cases in which securing a bloodless field is difficult, employing deep hypothermic circulatory arrest in patients who can safely use a cardiopulmonary bypass is an effective method to ensure a bloodless field and perform surgery safely.

## Declarations

### Informed consent

I have obtained consent from the patient to the paper.

### Acknowledgments

Not applicable.

### Disclosure statement

The authors declare that they have no competing interests.

### Author contributions

MSh, MA, MF, YH, and HW contributed to the success of this operation. KW, YT, YK, CI, MSu, HT, YS, and GK contributed to the manuscript drafting.

Critical review and revision: all authors.

Final approval of the article: all authors.

Accountability for all aspects of the work: all authors.
